# The “echo-free space” technique: a safe and reliable method for endoscopic ultrasound scope insertion

**DOI:** 10.1055/a-2062-5718

**Published:** 2023-05-04

**Authors:** Shunsuke Omoto, Mamoru Takenaka, Tomohiro Fukunaga, Kota Takashima, Yoriaki Komeda, Seok Jeong, Masatoshi Kudo

**Affiliations:** 1 Departments of Gastroenterology and Hepatology, Kindai University Faculty of Medicine, Osaka-Sayama, Japan; 2 Division of Gastroenterology Department of Internal Medicine, Inha University Hospital, Inha University School of Medicine, Incheon, South Korea

In recent years, the applications of endoscopic ultrasound (EUS) have expanded greatly, not only as an observational tool but also as a treatment procedure such as with EUS-guided fine-needle aspiration (FNA) and EUS-guided drainage 1 2 3 4 .

Both EUS-FNA and EUS-guided drainage require a linear-type EUS scope, which can be challenging to insert into the pylorus. As the EUS scope is a side-viewing scope, the tip does not face the pylorus, which may result in damage to the stomach wall and lead to unsuccessful insertion into the pylorus if the scope is advanced with reference to the endoscopic image ( Fig. 1 ). For this reason, inserting an EUS scope into the pylorus can be challenging for trainees learning this procedure 5 . Even expert EUS sonographers often experience pyloric insertion difficulties, resulting in repeated insertion attempts and gastric mucosal damage.

**Fig. 1:**
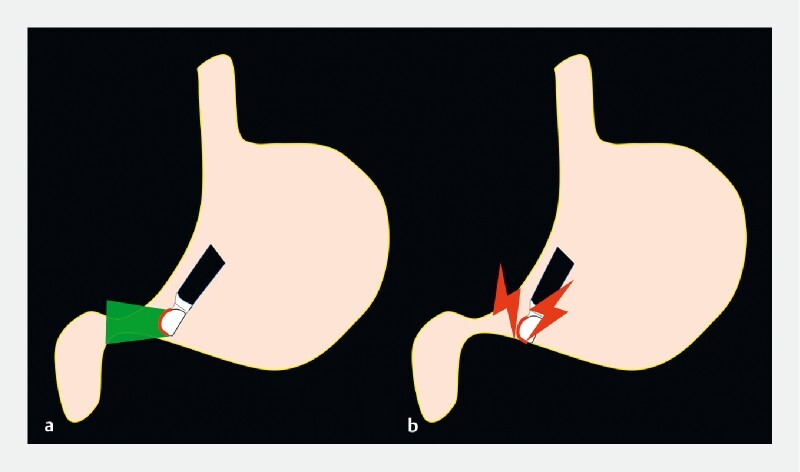
When the endoscopic ultrasound scope is advanced with reference to the endoscopic image ( **a** ), the tip of the scope does not face the pylorus, resulting in possible damage to the stomach wall and unsuccessful insertion into the pylorus ( **b** ).

We have therefore developed a safe and reliable method for inserting an EUS scope into the pylorus, which utilizes the “echo-free space” method.

First, advance the EUS scope in front of the pyloric ring and search for the lumen of the duodenal bulb, which is depicted as an “echo-free space” on EUS ( Fig. 2 a ). Next, search for the site where the continuity of the intestinal wall adjacent to the echo-free space is interrupted ( Fig. 2 b ). As this break would be the pyloric ring, aligning the tip of the scope with the discontinuity on the EUS screen will lead to successfully inserting the EUS scope into the pylorus ( Fig. 2 c , Video 1 ).

**Fig. 2:**
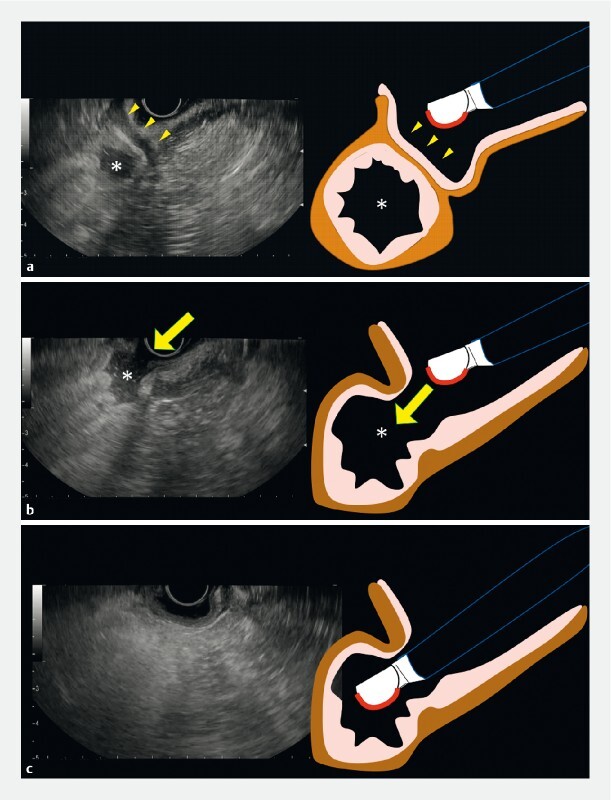
The “echo-free space” method. **a** First, advance the endoscopic ultrasound (EUS) scope in front of the pyloric ring (arrows) and search for the lumen of the duodenal bulb, which is depicted as an echo-free space (*) on EUS. **b** Next, search for the site where the continuity of the intestinal wall adjacent to the echo-free space (*) is interrupted (arrow). This part would be the pyloric ring. **c** Aligning the tip of the scope with the discontinuity on the EUS screen will lead to successful insertion of the EUS scope into the pylorus.

**Video 1** A safe and reliable method for inserting an endoscopic ultrasound scope into the pylorus, utilizing the “echo-free space” method.


This method, guided only by the EUS image and not the endoscopic image, is a safe and reliable method for inserting the EUS scope into the duodenal bulb. It can be a useful technique not only for trainees but also for experienced EUS sonographers in cases where insertion is difficult.

Endoscopy_UCTN_Code_CCL_1AF_2AZ

## Notes

Endoscopy E-Videos

https://eref.thieme.de/e-videos
*E-Videos* is an open access online section of the journal *Endoscopy* , reporting on interesting cases and new techniques in gastroenterological endoscopy. All papers include a high-quality video and are published with a Creative Commons CC-BY license. Endoscopy E-Videos qualify for HINARI discounts and waivers and eligibility is automatically checked during the submission process. We grant 100% waivers to articles whose corresponding authors are based in Group A countries and 50% waivers to those who are based in Group B countries as classified by Research4Life (see: https://www.research4life.org/access/eligibility/ ). This section has its own submission website at https://mc.manuscriptcentral.com/e-videos
